# IAA-producing bacteria from the rhizosphere of chickpea (*Cicer arietinum* L.): Isolation, characterization, and their effects on plant growth performance

**DOI:** 10.1016/j.heliyon.2024.e39702

**Published:** 2024-10-22

**Authors:** Debebe Landina Lata, Oumer Abdie, Yayis Rezene

**Affiliations:** a Department of Biotechnology, College of Natural and Computational Science, Wolkite University, Wolkite, Ethiopia; b Molecular Biotech Laboratory, Southern Agricultural Research Institute, Hawassa, Ethiopia

**Keywords:** Bio-inoculants, Chickpea, Indole-3-acetic acid, Molecular analysis, PGPR, Rhizosphere

## Abstract

Indole-3-acetic acid (IAA), a crucial plant hormone, regulates diverse physiological processes. This study aimed to isolate and characterize IAA-producing bacteria from the chickpea (*Cicer arietinum* L.) rhizosphere and evaluate their effects on plant growth. From 54 rhizosphere samples, 118 bacteria (designated as GAC) were isolated and screened for IAA production using a Salkowski colorimetric assay, and Bergey's manual was used for biochemical identification. Isolates were grown under various conditions and *in vitro* screened for their growth promotion traits. A PCR investigation was performed for IAA and nitrogen-fixing genes, and evaluated for greenhouse conditions. Among them, 27 isolates produced IAA, with eight high producers selected. Morphological and biochemical identification classified the six isolates as *Pseudomonas* and the other two as *Bacillus*. Optimal conditions for IAA production were observed at 500 μg/ml tryptophan, 35 °C, and pH 7.0. A 48-h incubation was ideal for IAA production, except for GAC-34 and GAC-73, which required 72 h. All the isolates achieved optimal IAA levels with tryptone and sucrose as nitrogen and carbon sources, respectively. Moreover, all isolates showed nitrogen fixation ability, and the six isolates exhibited phosphate solubilization. PCR confirmed the amplification of *nifH* (300 bp), *nifK* (360 bp), and *ipdC* (1170 bp) genes. Greenhouse experiments demonstrated that eight selected isolates significantly enhanced chickpea growth parameters (p < 0.001). These findings suggest that these IAA-producing bacteria have the potential to be used as biofertilizers to improve crop productivity, although further molecular identification and field studies are required.

## Highlights

• *Pseudomonas* and *Bacillus* isolates used as high IAA-producing isolates.
• Greenhouse experiments revealed significant enhancement in chickpea growth parameters.
• PCR confirmed the presence of *nifH, nifK,* and *ipdC* genes.
• 500 μg/ml tryptophan considered as optimal IAA production conditions.

## Graphical abstract

**Figure:**
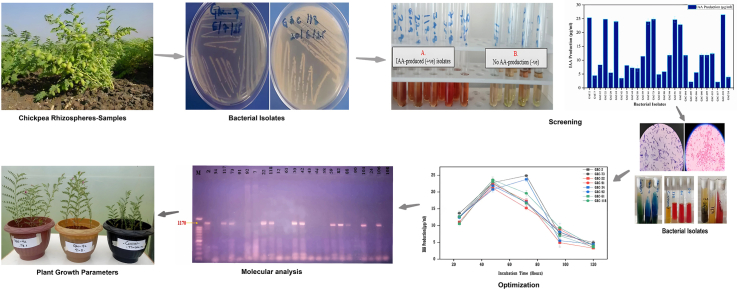

## 1 Introduction

The growing global population, projected to reach 9.8 billion by 2050, has heightened the need for increased food production, particularly in developing countries [1]. Ethiopia, with its diverse agroecologies, has the potential to meet this demand through varied farming methods, especially with grain legumes like chickpea (*Cicer arietinum* L.)*,* a significant pulse crop known for its high protein content. The Amhara and Oromia regions account for about 93 % of Ethiopia's total chickpea production, making it a critical component of the nation's agriculture [2,3].

To meet food demands, farmers have increasingly relied on agrochemicals to boost crop yields [4]. However, the extensive use of these chemicals has led to environmental pollution, reduced soil fertility, and threats to human health [5]. In response to the damage caused by agrochemicals, scientists are exploring microorganism-based approaches like Plant Growth Promoting Rhizobacteria (PGPR). These bacteria can enhance plant growth through various mechanisms, including the production of phytohormones, nitrogen fixation, and phosphate solubilization [6]. PGPR encompasses a wide range of bacterial genera, with species like *Pseudomonas* and *Bacillus* being particularly noted for their ability to promote plant growth by producing indole-3-acetic acid (IAA) and other beneficial compounds. It is estimated that around 80 % of bacteria isolated from the rhizosphere can produce IAA, a key plant hormone [7].

IAA is a naturally occurring auxin essential for plant growth. Microbes synthesize IAA through both tryptophan-dependent and independent pathways, with L-tryptophan being the most efficient precursor [8]. Among these pathways, the indole-3-pyruvic acid (IPyA) pathway is particularly important for IAA biosynthesis in bacteria [12]. The IpdC gene plays a crucial role in this process by encoding the enzyme that converts IPyA to IAA [9]. IAA production by rhizospheric bacteria influences various stages of plant growth, including seed germination, root and shoot development, and overall biomass [10].

Recent advancement in PGPR underscores their vital role in organic farming, sustaining soil fertility, and promoting plant growth [11]. Scientific interest in the potential of PGPR in agriculture has been steadily increasing, providing an attractive alternative to costly synthetic fertilizers and pesticides [5]. Despite the crucial role of IAA as a plant hormone regulating various physiological processes, there is a significant knowledge gap regarding how IAA-producing bacteria precisely promote plant growth and function as effective biofertilizers, particularly in the context of Ethiopia. Limited laboratory or field studies have explored the potential of PGPR and bioinoculants in promoting plant growth. The scarcity of information on the diversity, prevalence, and characteristics of these bacteria hinders the development of sustainable agricultural practices, and the specific impact of IAA-producing bacteria on plant growth performance remains unclear, lacking well-defined mechanisms and outcomes of bacterial-plant interactions, especially in Ethiopia. Therefore, the objective of this study was to isolate and characterize IAA-producing bacteria from the rhizosphere of chickpea (*Cicer arietinum* L.) and to evaluate their effects on plant growth performance in selected districts of the Gurage Zone.

## 2 Materials and methods

### 2.1 Study sites and time

**Fig. 1:**
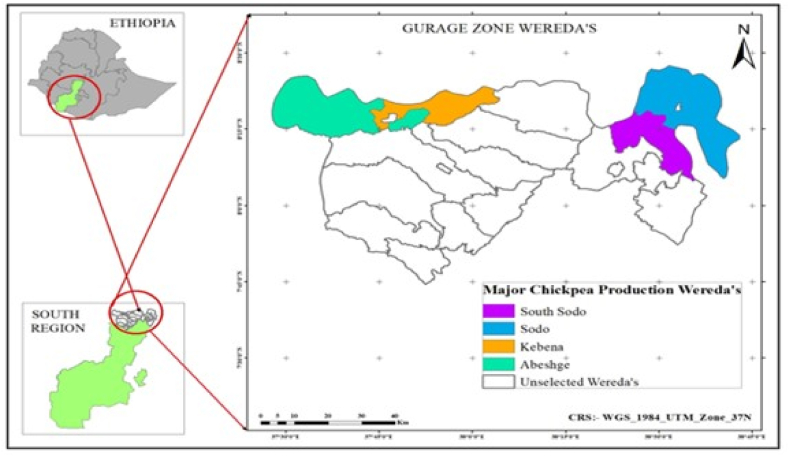
Map of chickpea rhizosphere samples collection sites/area.

The experiment was conducted in the Molecular Biotechnology Laboratory, Department of Biotechnology, Wolkite University, Wolkite, and Southern Agricultural Research Institute (SARI), Molecular Biotechnology Laboratory, Hawassa, Ethiopia. The study sites of this research comprise four districts of the Gurage Zone: Abeshige, Kebena, South Sodo, and Sodo **(**Fig. 1). These areas were selected due to their significant chickpea production and lack of prior chickpea rhizosphere bacteria inoculation. The rhizosphere samples, including pink-colored nodules and soil from the roots and rhizosphere of a healthy standing plant, were collected between December 2022 and January 2023. This collection occurred during the late flowering stage.

### 2.2 Sample collection and sample size determination

A total of 54 samples (24 root nodules and 30 rhizosphere soil) of cultivated chickpea local varieties were collected during the flowering stage from four selected sites. A completely randomized design (CRD) based experimental procedure with three replications was used as a study design to evaluate the efficiency of IAA-producing bacteria. At each sampling site, the late flowering and early pod setting stages root nodule samples were detached from roots (cut the root 0.5 cm on each side of the nodule). Triplicates of chickpea root nodules from young, healthy seedlings were promptly placed into sealed vials with silica gel [49]. Approximately 50 g of soil was collected at a depth of 0–15 cm from each sampling site using a sterilized spatula and transferred to an ethanol-sanitized (70 %) polyethylene plastic bag [4]. Next, the chickpea rhizosphere samples were transported to Wolkite University's Molecular Biotechnology Laboratory, where it was kept isolated at 4 °C. The Ethiopian Institute of Biodiversity (EBI) provided chickpea seed accession 41209.

### 2.3 Bacteria isolation

For bacterial isolation, soil samples were collected from a depth of 0–15 cm using a sterilized spatula and transferred to ethanol-sanitized polyethylene bags. Bacterial isolation followed the serial dilution plate method [13]. One gram of soil was mixed with 10 mL of saline solution (0.85 % NaCl), then serially diluted from 10^−1^ to 10^−7^. A 0.1 mL sample from each dilution was aseptically transferred to sterile Petri plates. *Pseudomonas* species were isolated on King's B agar, while *Bacillus* species were isolated on nutrient agar, with plates incubated for 48 h at 28 ± °C. Root nodules were sterilized using 70 % ethanol and 5 % sodium hypochlorite (NaOCl), rinsed with sterile water, and then crushed in saline solution (0.85 % NaCl) inside the laminar airflow cabinet. The resulting suspension was streaked on respective media and incubated for 48 h at 28 ± 2 °C [7]. Single colonies were purified through repeated re-streaking. All purified isolates were designated as GAC (Gurage Auxin Chickpea) with unique numbers (e.g., 1, 2, 3, … 118), preserved on slants, and stored at 4 °C.

### 2.4 Screening of bacterial isolates for IAA-production

The production of IAA by the bacterial isolate was assessed using the method described by Ehmann [14]. The selected isolate was cultured in yeast extract mannitol broth (YEMB) supplemented with 500 μg/ml L-tryptophan, with the experiment conducted in triplicate. The culture was incubated at 28 ± 2 °C for 3 days. After incubation, approximately 1.5 mL of the culture was transferred into sterile Eppendorf tubes and centrifuged at 10,000 rpm for 10 min. IAA production was then measured by adding the Salkowski reagent to the bacterial culture supernatant, and the absorbance was recorded at 530 nm using a spectrophotometer.

### 2.5 Morphological and biochemical identification of IAA-producing bacterial isolates

The IAA-producing bacterial isolates were physically identified using colonial and microscopic morphology. Biochemical tests were performed following procedures in Bergey's Manual of Systematic Bacteriology [15]. Characterization of cultures included gram staining and morphological assessment (colony color, shape, and size) after 24 h on LB agar at 28 ± 2 °C. Biochemical tests covered catalase, starch hydrolysis, citrate utilization, methyl red, vogues Proskauer, and triple sugar iron tests [16].

### 2.6 Optimization of IAA production by bacterial isolates

To maximize the synthesis of IAA, bacteria isolates were grown under various conditions, including L-tryptophan (100, 200, 300, 400, 500, and 600 μg/ml) concentration [17], incubation times (24, 48, 72, 96, and 120 h) [18], pH (5, 6, 7, 8, and 9) [19], temperature (25, 30, 35, 37, and 45 °C) [20], and carbon sources (1 %, w/v) (sucrose, fructose, glucose, and dextrose) and nitrogen sources (1 %, w/v) (peptone, tryptone, yeast extract, and beef extract) on the yeast extract mannitol broth media [19]. The IAA production was estimated at 530 nm using the Salkowski reagent.

### 2.7 *In vitro* screening of IAA-producing bacteria for growth production traits

#### 2.7.1 Phosphate solubilization activity

In the assessment of IAA-producing bacterial isolates, their phosphate solubilizing activity was screened *in vitro* using Pikovskaya's medium following the method outlined by Yadav [21]. Pinpoint inoculation was performed on agar plates, and the medium was autoclaved at 121 °C for 15 min. The plates were then incubated at 30 °C for 2–3 days. Phosphate solubilization by bacteria was confirmed by the formation of a clear zone around the colony. The phosphate solubilization index (PSI) of the isolate was determined by calculating the ratio of the halo zone to the colony size.

PSI = [colony diameter (cm) + halo zone diameter (cm)]/colony diameter (cm)

#### 2.7.2 Nitrogen fixation activity

The nitrogen-fixing activities of IAA-producing bacterial isolates were visually detected using a glucose-nitrogen-free mineral medium (G-NFMM) (HiMedia) with bromothymol blue (BTB) solution as a color indicator [22]. Pure bacterial colonies were streaked and cultured for 3–5 days at 30 °C. After incubation, the ability of bacteria isolates to fix nitrogen was determined by the medium's color shifting from greenish blue to dark blue or yellow. When the generation of acid changes from green to blue or yellow, it means that the bacterial isolates are actively fixing nitrogen [23].

### 2.8 Molecular analysis of IAA and nitrogen-fixing related genes

#### 2.8.1 Genomic DNA extraction

Genomic DNA extraction was carried out using a modified method of Andleeb et al. [24] involving phenol-chloroform. The bacterial isolates were transferred to 1.5 mL Eppendorf tubes and centrifuged at 8000 rpm for 5 min. The resulting pellet in each tube was resuspended in 250 μL of Tris-EDTA (TE) buffer, followed by the addition of 30 μL of 10 % Sodium Dodecyl Sulfate (SDS) and 3 μL of 20 mg/mL proteinase K. The mixture was gently pipetted to mix well and incubated for 1 h at 37 °C. An equal volume of phenol: chloroform was added, mixed well by inverting the tube until the phases were completely mixed, and then centrifuged at 12,000 rpm for 10 min. The upper aqueous phase was transferred into a new tube, and 1/10th volume of sodium acetate and 0.6 volume of isopropanol were added. The mixture was gently mixed until the DNA precipitated. The tubes were then spun at 8000 rpm for 5 min. The DNA pellet was washed with 1 mL of 70 % ethanol, placed at 4 °C for 15 min, and centrifuged at 8000 rpm for 5 min. The supernatant was discarded, and the DNA pellet was air-dried. To expedite drying, it was drained well onto a Kimwipe for 10 min. The DNA was then resuspended in 100 μL of TE buffer. DNA was stored at 4 °C for short-term storage and at −20 °C for long-term storage. The concentration of each DNA sample was measured using spectrophotometric analysis.

#### 2.8.2 PCR amplification of genes

PCR amplification of nitrogen-fixing and IAA-related genes was performed using the PCR primers: for the nifH gene, nifHF-5′-GGCAAGGGCGGTATCGGCAAGTC-3′ and nifHR-5′-CCATCGTGATCGGGTCGGGATG-3′ at 61 °C (300 bp) [23], the nifK gene, nifKF-5′-CCTGGATGACCGAAGACGC-3′ and nifKR-5′-GGTGCCGCCTTCATACAT-3′ at 59.95 °C (360 bp) [25], and the ipdC gene, ipdCF-5′-AGAAGTCGCCGGTCGTCGTCAT-3′ and ipdCR-5′-CCGCCAGTCGTCCAGGTCATTG-3′ at 59.97 °C (1170 bp) [12]. The amplification conditions were optimized to achieve a high level of consistency in band patterns, utilizing agarose gel electrophoresis with a concentration of 1.3 % (w/v). PCR amplification was carried out in 25 μL volume, including 0.5 μL of 10 mmol/l dNTPs, 2.5 μL of 10 × buffer (MgCl_2_+), 0.2 μL of 5 U/μL Taq polymerase, 1 μL of 10 μmol/L of each gene primer, and 2 μL of 50 ng/μL genomic DNA. Nuclease-free water was used to achieve 25 μL of the total volume. The PCR conditions were as follows: an initial denaturation at 94 °C for 5 min, followed by 35 cycles of denaturation for 20 s at 94 °C, annealing for 30 s depending on the annealing temperature of the gene primers, elongation for 1 min at 72 °C, a final extension for 10 min at 72 °C, and a 4 °C holding step for a maximum of 24 h.

#### 2.8.3 Agarose gel electrophoresis

The amplified DNA was separated by electrophoresis in a 1.3 % (w/v) agarose gel run in 1 × TAE buffer, using a Gel Doc UV transilluminator (Bio-Rad), according to the methods by Ratna & Meliah [26], with slight modifications. The horizontal gel electrophoresis was carried out at a voltage of 90 V for 90 min to 2 h till DNA fragments were well migrated. The resulting amplicons were subjected to agarose gel electrophoresis to visualize and confirm the presence and absence of the genes at the expected molecular weight.

### 2.9 Greenhouse evaluation of IAA-producing isolates for growth promotion

#### 2.9.1 Inoculum preparation

The eight highest IAA-producing bacterial isolates were selected for greenhouse evaluation in the Department of Biotechnology, Wolkite University. Flasks with a capacity of 250 ml were selected and filled with 150 ml of King's B and nutrient broth media. They were sterilized using the steam sterilization process and cooled in the hood. Then, a single colony of pure bacteria isolates was grown in 25 ml of the respective medium at 28 ± 2 °C for 48 h in the shaking incubator by setting the speed to 150 rpm. After 48 h of incubation, the standard concentration was adjusted to 1x10^9^.

#### 2.9.2 Plant growth promotion in a pot experiment

The effects of eight selected high IAA-producing bacteria on plant growth in the target test were evaluated in pot experiments. The experiment included non-inoculated soil (control) and five seeds per pot treated with *Pseudomonas* and *Bacillus* isolates. Pots were kept under controlled conditions until the end of the experiment [21]. Thus, nine treatments (eight bacterial isolates and one control group) were used for the pot experiment, each with three replications.

Test plants were sterilized by washing with 95 % ethanol (v/v) for 30 s, followed by soaking in 5 % bleach (v/v) for 3 min, and rinsing with sterile distilled water five times to remove excess bleach [27]. The surface-sterilized seeds were soaked in a broth culture of *Bacillus* and *Pseudomonas* amended with sucrose (0.2 %) to facilitate bacterial adherence to the seeds. Control seeds were soaked in distilled water for 30 min and dried for 15 min.

Plastic pots with a capacity of 3 kg were filled with 2 kg of sterilized sandy loam soil (sterilized at 121 °C for 15 min). Under greenhouse conditions, five seeds were planted in each pot, with three replications for each treatment. The control treatment was maintained without bacterial isolates. To meet the IAA needs of the plants, 30 ml of each treatment with a standard concentration of 1x10^9^ CFU/ml was added to the pots as a soil drench in equal amounts seven days later when the first and second leaves appeared. After 45 days of germination, the plants were gently removed from the pots with their roots intact and washed with distilled water to eliminate any remaining soil. Key growth parameters, including shoot length, root length, and the fresh and dry weight of the whole plant, were measured as indicators of plant growth and development [28].

### 2.10 Statistical analysis

Agronomic parameters (i.e., plant shoot height, plant shoot fresh and dry weight and root length, root fresh and dry weight) from a pot experiment underwent statistical analysis using One-Way ANOVA at 0.05 using SPSS version 27. Optimization parameters were schemed using Origin Pro 2021. The relationship between agronomic parameters was assessed through Pearson correlation coefficient analysis.

## 3 Results

### 3.1 Isolation and screening of bacterial isolates for IAA production

**Fig. 2:**
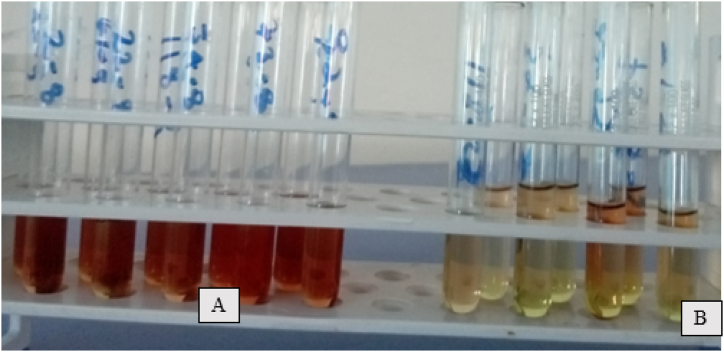
IAA production by bacterial isolates; (A): pink color positive result and (B): yellow color negative result.

A total of 118 bacterial isolates were obtained, and among them, 74 isolates were isolated from the soil rhizosphere and 44 isolates were from the root nodule sample. Over 50 (42.37 %) isolates of *Bacillus* spp*.* and 68 (57.63 %) isolates of *Pseudomonas* spp. were obtained using the appropriate media growth. Out of 118 isolates, only 27 exhibited a positive reaction by developing a pink or pink-red color when reacted with Salkowski's reagent, which indicates a positive result for IAA production based on the qualitative determination (Fig. 2).

**Fig. 3:**
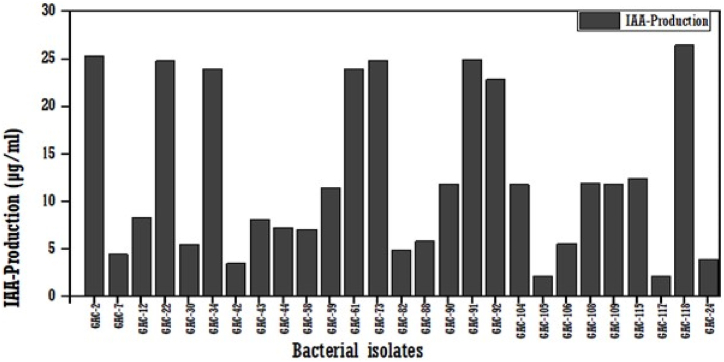
IAA production by bacteria isolates from chickpea rhizosphere samples.

All 27 isolates produced IAA in the range of 2.15 μg/ml to 26.47 μg/ml **(**Fig. 3**).** Among them, 8 isolates, GAC-2 (25.38 μg/ml), GAC-22 (24.83 μg/ml), GAC-34 (20.01 μg/ml), GAC-61 (23.95 μg/ml), GAC-73 (24.86 μg/ml), GAC-91 (24.95 μg/ml), GAC-92 (22.88 μg/ml), and GAC-118 (26.47 μg/ml) produced the highest concentration of IAA, and the lowest concentrations were exuded by GAC-117 (2.15 μg/ml) and GAC-105 (2.21 μg/ml).

### 3.2 Morphological and biochemical identification of IAA-producing bacterial isolates

**Fig. 4:**
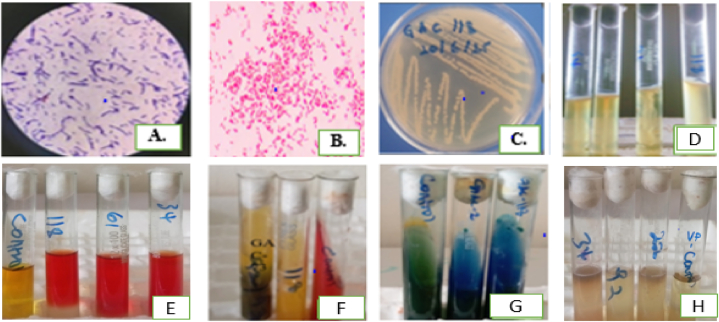
Morphological and biochemical characterization of bacterial isolates. (A): gram-positive isolate; (B): gram-negative isolate; (C): GAC-118 isolate on Kings B-medium; (D): positive motility test; (E): positive methyl red test; (F): positive triple sugar iron test; (G): positive citrate utilization; and (H): negative Vogues-Proskauer's test.

Based on morphological and biochemical reactions, two isolates (GAC-2 and GAC-73) were *Bacillus* species, and the remaining six isolates were examined and named *Pseudomonas* species (Fig. 4; Table 1).

**Table 1:** Morphological and biochemical characterization of IAA-producing bacterial isolates.

Biochemical characterization	Isolates code							
	GAC-2	GAC-22	GAC-34	GAC-61	GAC-73	GAC-91	GAC-92	GAC-118
Gram's reaction	+	–	–	–	+	–	–	–
Shape	Rod	Rod	Rod	Rod	Rod	Rod	Rod	Rod
Color	Whitish yellow	Creamy White	Yellow	Yellowish white	Whitish yellow	Whitish yellow	Creamy White	Yellow
Size	Small	Medium	Medium	Medium	Medium	Medium	Medium	Small
Motility	Motile	Motile	Motile	Motile	Motile	Motile	Motile	Motile
Catalase	+	+	+	+	+	+	+	+
MR test	+	+	+	–	+	+	+	+
VP	+	–	–	–	–	–	–	–
Indole	–	–	–	–	–	–	–	–
Citrate utilization	+	+	+	+	+	+	+	+
Starch hydrolysis	+	+	–	–	+	+	+	–
Gelatin hydrolysis	+	+	+	+	–	–	–	+
Urease test	–	–	–	–	–	+	+	–
Oxidase	+	+	+	+	+	+	+	+
Gas production	–	+	+	–	+	+	–	+
H_2_S production	–	–	–	–	+	–	–	–
Glucose	+	+	+	+	+	+	–	+
Lactose	–	+	+	–	–	+	–	+
Sucrose	+	–	–	–	–	–	–	–
Similarity of Bacteria species	*Bacillus* sp.	*Pseudomonas* spp.	*Pseudomonas* sp.	*Pseudomonas* sp.	*Bacillus* sp.	*Pseudomonas* sp.	*Pseudomonas* sp.	*Pseudomonas* spp.

(+) = Gram-positive bacteria; (−) = Gram-negative bacteria; GAC = Gurage Auxin Chickpea; VP=Vogues-Proskauer's; MR = Methyl red; H_2_S=Hydrogen sulfide; (+) = Production; (−) = No production.

### 3.3 Optimization of IAA production by bacterial isolates

#### 3.3.1 Effect of tryptophan concentration on IAA production

**Fig. 5:**
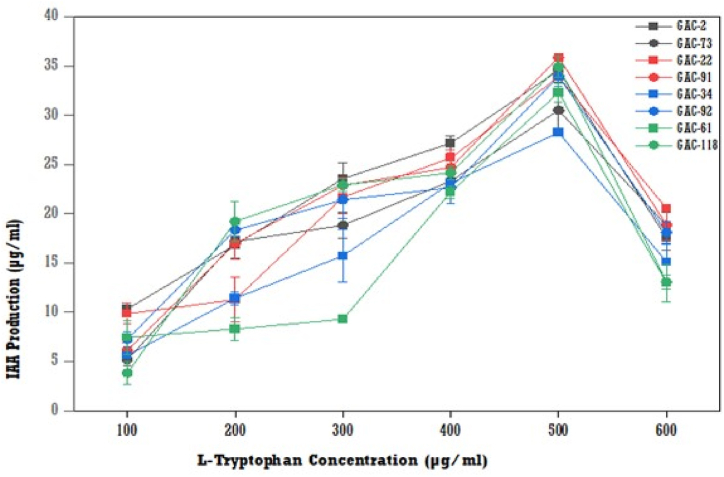
Effect of different L-tryptophan concentrations on IAA production by bacterial isolates (means (n = 3) ± standard deviation).

Based on the spectrophotometric analysis, the results showed that increasing production of IAA along with L-tryptophan increased concentrations up to 500 μg/ml and then started to decrease production of IAA for all isolates (Fig. 5). The maximum IAA production was observed on the medium added with 500 μg/ml L-tryptophan for all the isolates. The highest IAA was produced on isolate GAC-2 with 34.56 μg/ml at 500 μg/ml of L-tryptophan concentration, followed by isolate GAC-91 (33.88 μg/ml) and isolate GAC-118 (33.37 μg/ml).

#### 3.3.2 Effect of incubation period on IAA production

**Fig. 6:**
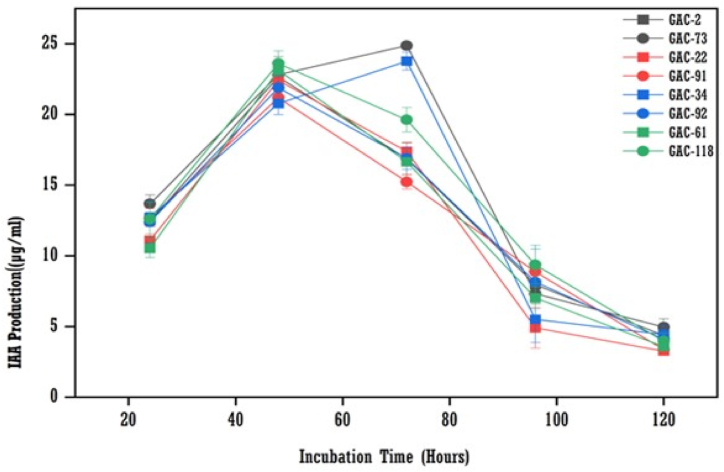
The effect of the incubation periods on IAA production by bacterial isolates (means (n = 3) ± standard deviation).

The production of IAA by the isolates began 24 h after incubation. Maximum IAA production was reached after 48 h for some isolates (GAC-2, GAC-22, GAC-61, GAC-91, GAC-92, and GAC-118), and after 72 h for remaining isolates (GAC-34 and GAC-73). The bacterial isolates then reached the stationary phase, with production decreasing after 96 and 120 h (Fig. 6). The maximum IAA production was observed after 72 h of incubation (24.88 μg/ml) by the GAC-73 isolate, followed by GAC-22 after 48 h of incubation (24.46 μg/ml).

#### 3.3.3 Effect of pH values on IAA production

**Fig. 7:**
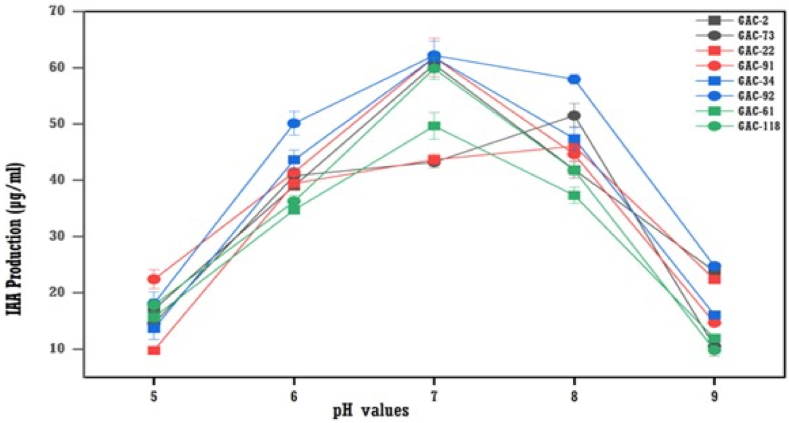
The effect of the different pH values on IAA production by bacteria (means (n = 3) ± standard deviation).

IAA production results after incubation showed that pH 7 and pH 8 were the optimum pH for IAA production for all bacterial isolates (Fig. 7). The maximum production of IAA was obtained on a medium with pH 7, followed by pH 8 and pH 6. The highest IAA production was at 61.85 μg/ml by GAC-91 followed by GAC-188 at 60.62 μg/ml at pH 7.

#### 3.3.4 Effect of temperature on IAA production

**Fig. 8:**
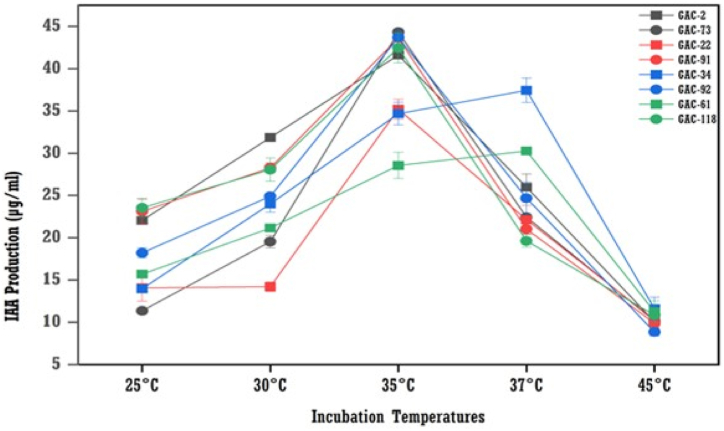
The effect of different temperatures values on IAA production by bacterial isolates (means (n = 3) ± standard deviation).

The results showed that 35 °C was suitable for six bacterial isolates for maximum IAA production, except GAC-34 and GAC-61 at 37 °C (Fig. 8). The GAC-118 isolate yielded the maximum IAA production at 52.89 μg/ml. This was followed by GAC-91 at 45.89 μg/ml and GAC-73 at 44.32 μg/ml, both at 35 °C. At 37 °C, GAC-61 produced 35.66 μg/ml and GAC-34 produced 34.94 μg/ml**.**

#### 3.3.5 Effect of carbon sources on IAA production

**Fig. 9:**
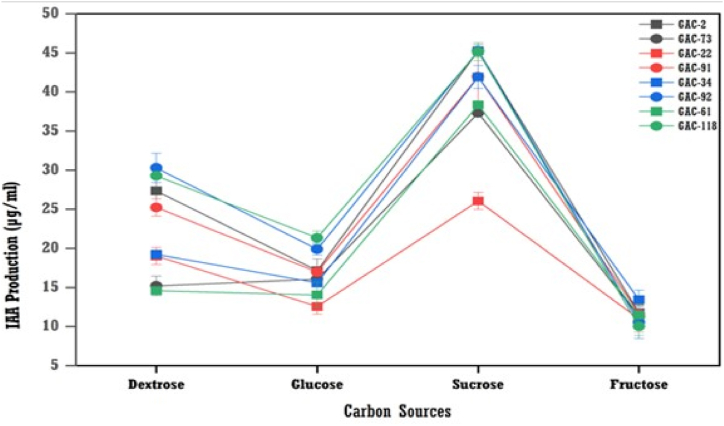
The effect of carbon sources concentration on IAA production by bacterial isolates (means (n = 3) ± standard deviation).

The most suitable carbon source for the highest production of IAA by bacterial isolates was sucrose, followed by dextrose, glucose, and fructose (Fig. 9). The GAC-2 isolate produced the highest amount of IAA at 45.28 μg/ml with sucrose, followed by the GAC-118 and GAC-91 isolates, which reached 44.89 μg/ml and 44.01 μg/ml, respectively. On the other hand, the GAC-118, GAC-2, GAC-92, and GAC-91 produced higher IAA (28.13 μg/ml, 27.35 μg/ml, 26.38 μg/ml, 26.35 μg/ml) under amended with dextrose.

#### 3.3.6 Effect of nitrogen sources on IAA production

**Fig. 10:**
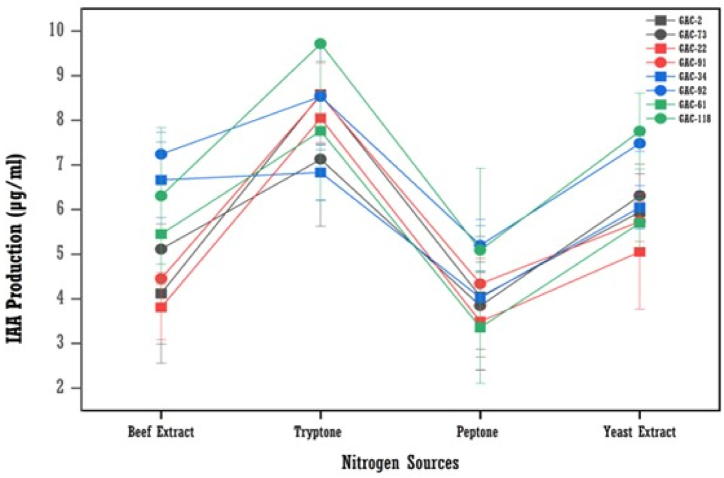
The effect of nitrogen sources concentration on IAA production by bacterial isolates (means (n = 3) ± standard deviation).

Results obtained showed that tryptone was the best nitrogen source for the maximum production of IAA for tested bacterial isolates, followed by yeast extract, beef extract, and peptone (Fig. 10). The optimum production of IAA was obtained in tryptone media, which was 9.23 μg/ml by the GAC-91 isolate, followed by the GAC-22 and GAC-2 isolates with 9.01 μg/ml and 8.58 μg/ml, respectively. The lowest IAA production was obtained at 2.81 μg/ml in peptone media for the GAC-34 isolate.

### 3.4 Screening of IAA-producing isolates for their plant growth promoting traits

#### 3.4.1 Phosphate solubilization

Two *Bacillus* isolates (GAC-2 and GAC-73) exhibited a clear halo zone of phosphate solubilization on Pikovaskaya's agar plate. The GAC-2 isolate exhibited a 4.0 mm solubilization zone, while GAC-73 showed a 5.0 mm solubilization zone. Among six *Pseudomonas* isolates, four formed clear halo zones, indicating phosphate solubilization on Pikovaskaya's agar plates. However, GAC-34 and GAC-92 did not exhibit clear halo zones on Pikovaskaya's agar plates. Among them, the GAC-118 isolate detected the highest clear zone at 11.00 mm followed by GAC-91 (8.5 mm).

#### 3.4.2 Nitrogen fixation

After incubation, GAC-91, GAC-73, and GAC-61 showed nitrogen fixation by producing ammonia, changing the medium color to distinctly blue from greenish blue. The remaining isolates (GAC-2, GAC-22, GAC-34, GAC-92, and GAC-118) exhibited nitrogen fixation by changing the medium color to yellow due to acid production from sugar utilization. Many nitrogen-fixing bacteria produce acids during this process.

### 3.5 Molecular analysis of genes

**Fig. 11:**
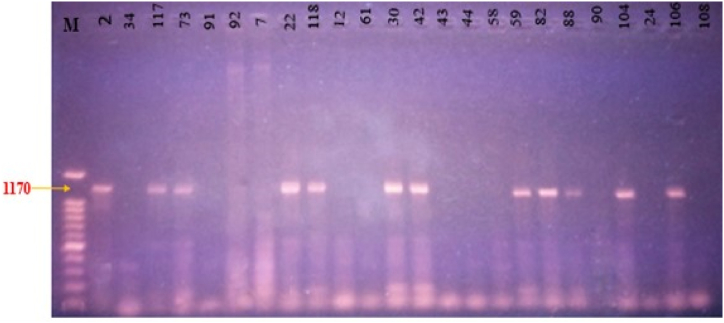
Gel electrophoresis of *ipdC* (1170 bp) gene amplified by PCR. Lane name with respective isolates; *M-(100 bp ladder), 2–(GAC-2), 34-(GAC-34), 117-(GAC-117), 73-(GAC-73), 91-(GAC-91), 92-(GAC-92), 7-(GAC-7), 22-(GAC-22), 118-(GAC-118), 12-(GAC-12), 61-(GAC-61), 30-(GAC-30), 42-(GAC-42), 43-(GAC-43), 44-(GAC-44), 58-(GAC-58), 59-(GAC-59), 82-(GAC-82), 88-(GAC-88), 90-(GAC-90), 104-(GAC-104), 24-(GAC-24), 106-(GAC-106), and 108-(GAC-108).*

**Fig. 12:**
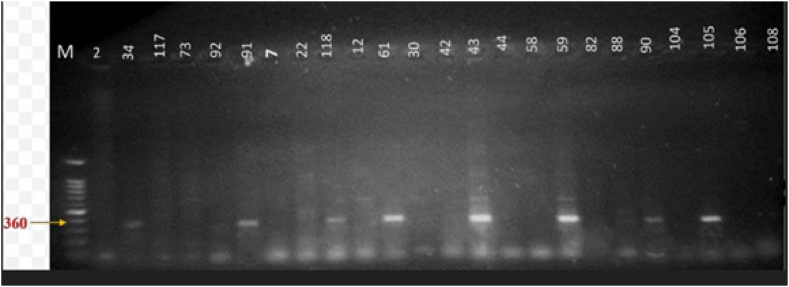
Gel electrophoresis of *nifK* (360 bp) gene amplified by PCR. Lane name with respective isolates; *M-(100 bp ladder), 2–(GAC-2), 34-(GAC-34), 117-(GAC-117), 73-(GAC-73), 92-(GAC-92), 91-(GAC-91), 7-(GAC-7), 22-(GAC-22), 118-(GAC-118), 12-(GAC-12), 61-(GAC-61), 30-(GAC-30), 42-(GAC-42), 43-(GAC-43), 44-(GAC-44), 58-(GAC-58), 59-(GAC-59), 82-(GAC-82), 88-(GAC-88), 90-(GAC-90), 104-(GAC-104), 105-(GAC-105), 106-(GAC-106), and 108-(GAC-108).*

Based on the PCR amplification, only 12 isolates (GAC-2, GAC-117, GAC-73, GAC-22, GAC-118, GAC-30, GAC-42, GAC-59, GAC-82, GAC-88, GAC-104, and GAC-106) possessed the ipdC gene. An 1170 bp DNA fragment found on the gel electrophoresis served as confirmation of this (Fig. 11). The product of the expected size (1170 bp) indicated the existence of an IAA-related gene in these isolated bacteria. The ipdC gene was absent from the other bacterial isolates. PCR amplification targeting the *nifK* gene revealed that 10 isolates (GAC-34, GAC-91, GAC-92, GAC-118, GAC-61, GAC-43, GAC-59, GAC-88, GAC-90, and GAC-105) produced an amplified fragment of approximately 360 bp, indicating the presence of the *nifK* gene (Fig. 12). In contrast, the nifK gene was not detected in the remaining isolates. These findings confirmed that the isolates carrying the nifK gene possessed the ability to fix nitrogen. The results from the nifH gene PCR amplification of the isolates revealed that only 8 isolates (GAC-34, GAC-118, GAC-73, GAC-91, GAC-7, GAC-117, GAC-30, and GAC-42) displayed successful amplification of the nifH gene. This was evidenced by observing the presence of DNA fragments of the expected size (around 300 bp) in the gel electrophoresis analysis. These findings suggest that these particular bacterial isolates harbor the genetic material responsible for nitrogen fixation. Conversely, none of the other isolates showed any presence of the *nifK* gene.

### 3.6 Greenhouse evaluation of IAA-producing isolates for growth promotion

**Fig. 13:**
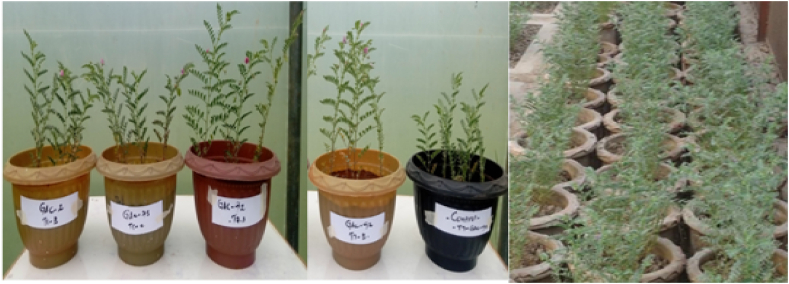
Plant growth promoting potential of IAA-producing isolates in pot experiment.

In the greenhouse experiment, target plants were carefully uprooted 45 days after sowing, and their growth parameters were measured (Fig. 13; Table 2). The greenhouse study showed that all the isolates led to a statistically significant increase (p ≤ 0.05) in chickpea growth compared to the control. GAC-118 had the greatest impact, boosting shoot height by 84 % (35.867 cm), shoot fresh weight by 253 % (8.373 cm), and shoot dry weight by 277 % (4.190 cm). GAC-91 and GAC-2 also showed notable increases: shoot heights of 68 % (32.710 cm) and 60 % (31.220 cm), fresh weights of 209 % (7.327 cm) and 206 % (7.263 cm), and dry weights of 250 % (3.883 cm) and 239 % (3.767 cm), respectively. GAC-22 decreased shoot height by 35 % (26.367 cm), and reduced both fresh and dry weights, while GAC-92 decreased shoot dry weight. Overall, GAC-118, GAC-91, and GAC-2 had the most significant positive effects. GAC-118 had the highest increase at 108 % (25.500 cm), followed by GAC-91 at 91 % (23.333 cm) and GAC-2 at 84 % (22.517 cm). GAC-22 decreased root length by 38 % (16.833 cm) compared to the un-inoculated control. For root fresh weight, GAC-61 had the highest increase at 144 % (4.360 cm), followed by GAC-73 at 131 % (4.127 cm) and GAC-188 at 125 % (4.013 cm). GAC-22 had the lowest increase at 57 %. For root dry weight, GAC-92 increased by 273 %, GAC-61 by 231 %, and GAC-73 by 228 %, while GAC-22 decreased by 62 % compared to the un-inoculated control.

**Table 2:** ANOVA analysis of IAA-producing bacterial isolates on chickpea growth parameters under greenhouse conditions. Values are means ± S.D.

Isolates code	Above ground growth parameter Below ground growth parameter					
	SH (cm)	SFW (g)	SDW (g)	RL (cm)	RFW (g)	RDW (g)
GAC-2	31.220 ± 2.368^abc^	7.26 ± 0.451^b^	3.767 ± 1.079^a^	22.517 ± 1.075^cb^	3.497 ± 0.542^bac^	2.073 ± 0.657^a^
GAC-22	26.367 ± 1.11^d^	3.41 ± 0.308^c^	1.487 ± 0.445^cd^	16.833 ± 0.585^d^	2.806 ± 0.930^c^	1.113 ± 0.225^bc^
GAC-34	29.567 ± 3.093^bcd^	5.66 ± 1.066^c^	2.380 ± 0.178^b^	21.833 ± 1.204^cb^	3.367 ± 0.351^bc^	1.896 ± 0.335^ba^
GAC-61	29.317 ± 2.004^bcd^	4.46 ± 0.386^d^	2.163 ± 0.155^cb^	21.033 ± 0.680^c^	4.360 ± 0.815^a^	2.277 ± 0.342^a^
GAC-73	29.167 ± 2.560^bcd^	4.31 ± 0.419^d^	2.067 ± 0.221^cb^	18.600 ± 2.007^d^	4.127 ± 0.499^ba^	2.250 ± 0.053^a^
GAC-91	32.710 ± 2.193^ba^	7.33 ± 0.495^b^	3.883 ± 0.021^a^	23.33 ± 1.528^b^	3.620 ± 0.720^bac^	2.197 ± 0.288^a^
GAC-92	28.400 ± 4.851^cd^	4.19 ± 0.066^cd^	1.880 ± 0.105^cd^	17.126 ± 0.424^d^	3.320 ± 0.030^bc^	2.563 ± 1.172^a^
GAC-118	35.867 ± 0.550^a^	8.373 ± 0.41^a^	4.190 ± 0.429^a^	25.500 ± 0.500^a^	4.013 ± 0.035^ba^	2.173 ± 0.153^a^
Control	19.463 ± 0.843^e^	2.37 ± 0.285^f^	1.110 ± 0.289^d^	12.240 ± 1.132^e^	1.787 ± 0.420^d^	0.687 ± 0.163^c^
CV	8.591	9.519	17.289	5.6783	16.516	25.939
LSD (5 %)	4.291	0.859	0.756	1.938	0.973	0.852
p	p < 0.001	p < 0.001	p < 0.001	p < 0.001	p < 0.001	p < 0.001

Means in a column followed by the same superscript letters are not significantly different at P ≤ 0.05.

SH = Shoot Height; SFW = Shoot Fresh Weight; SDW = Shoot Dry Weight; RL = Root Length; CV= Coefficients of variation; RFW = Root Fresh Weight: RDW = Root Dry Weight; LSD = Least significant differences of means (5 % level).

The ANOVA test showed significant increases (p ≤ 0.05) in all the agronomic parameters compared to the control. Pearson correlation analysis (Table 3) revealed strong and moderate positive correlations among parameters like shoot height, fresh and dry weight, root length, and root fresh and dry weight. The study's growth-promoting rhizobacteria were able to consistently and successfully influence all growth-related traits, including the production of IAA, as indicated by the strong and moderate positive correlations found among the agronomic parameters.

**Table 3:** Pearson correlation coefficients for agronomic parameters.

	Correlations					
	SH	SFW	SDW	RL	RFW	RDW
SH						
**SFW**	0.820^b^					
**SDW**	0.759^b^	0.906^b^				
**RL**	0.866^b^	0.911^b^	0.841^b^			
**RFW**	0.575^b^	0.461^b^	0.448^b^	0.579^b^		
**RDW**	0.655^b^	0.439^a^	0.501^b^	0.538^b^	0.578^b^	

SH = Shoot Height; SFW = Shoot Fresh Weight; SDW = Shoot Dry Weight; RL = Root Length; RFW = Root Fresh Weight: RDW = Root Dry Weight.

a correlation is moderately significant at 0.05.

b correlation is moderately significant at 0.001.

## 4 Discussion

### 4.1 Isolation and screening of bacterial isolates for IAA production

Phytohormones, or plant growth regulators, significantly impact plant growth [29]. Many rhizosphere species synthesize IAA, crucial for regulating growth and gene expression [22]. We have isolated and characterized *Pseudomonas* and *Bacillus* species that exhibit plant growth-promoting traits such as IAA production, nitrogen fixation, phosphate solubilization, and ammonia production. IAA production is linked to improved root growth, while nitrogen fixation and phosphate solubilization contribute to making nitrogen, mineral phosphates, and calcium available to plants [28]. Similar studies report bacterial IAA production from 20 mg/l to 90 mg/l with 0.5 g/l tryptophan [30]. Predominantly gram-negative, IAA producers include some gram-positive *Bacillus* strains [17,29]. Specific strains like *Pseudomonas putida UB1* [31]*,* Rhizobium *leguminosarum* strains [19], *and Bacillus siamensis* [20] also produce phytohormones. IAA production varies by species, culture conditions, substrate availability, and growth stage [18,30].

### 4.2 Optimization of IAA production by bacterial isolates

L-tryptophan, an essential amino acid, is crucial for various plant biological processes [17]. This study showed IAA production increased with L-tryptophan concentrations up to 500 μg/ml, with the highest IAA levels at this concentration for all bacterial isolates. This aligns with Shoukry et al. [19] and Bharucha et al. [31], who reported similar trends with L-tryptophan concentrations up to 5 mg/ml and 200 μg/ml, respectively. IAA production began after 24 h, peaking at 72 h, after which bacterial growth decreased. These findings match Shoukry et al. [19], who identified 48–72 h as the optimal incubation period, while Sadhana et al. [5] found 96–120 h optimal. The decline in IAA after 72 h may be due to IAA-degrading enzymes like IAA oxidase and IAA peroxidase [19].

The pH of production media is crucial for IAA-producing organisms. This study found the highest IAA production at pH 7, followed by pH 8, aligning with Shoukry et al. [19]. Bharucha et al. [31] reported maximum IAA production at pH 7.5 for *Pseudomonas putida* UB-1, while Chandra et al. [27] observed it at pH 9. Six isolates showed peak IAA production at 35 °C, and two at 37 °C, consistent with Chandra et al. [27] and Shoukry et al. [19].

The carbon source is crucial for metabolite production by microorganisms. This study found sucrose stimulated the highest IAA production, followed by dextrose, glucose, and fructose, with fructose resulting in the lowest IAA production. Sucrose's superior performance is due to its better bacterial utilization. Similar observations were reported by Chandra et al. [27] and Chouhan et al. [32]. Additionally, Bharucha et al. [31] demonstrated that bacteria grown on media with 1 % sucrose exhibit the highest IAA production.

Nitrogen is crucial for proteins, nucleic acids, and cell walls, impacting microbial growth and metabolism [32]. This study found tryptone medium resulted in the highest IAA production, followed by yeast extract, beef extract, and peptone. These findings align with Widawati & Suliasih [20], who reported *Bacillus siamensis* produced maximum IAA with tryptone as the nitrogen source.

### 4.3 Screening of IAA-producing isolates for their plant growth promoting traits

Nitrogen fixation is crucial for selecting potential PGPR. Bacterial isolates grown on G-NFM medium showed nitrogen fixation by producing ammonia [22], changing the medium color from greenish-blue to dark blue. Some isolates turned the medium yellow, indicating acid production from sugar utilization. The color change, due to bromothymol blue, suggests ammonium production, increasing medium pH [23]. These findings align with Tsegaye et al. [4] and Lwin et al. [45], who found nitrogen-fixing bacteria in Sorghum (*Sorghum bicolor*), and various rhizosphere soils, respectively.

Though phosphorus is abundant in soils, its low solubility makes it inaccessible to plants [22]. Researchers are exploring rhizobacteria's ability to solubilize phosphates to enhance plant growth and yield. Using phosphate-solubilizing PGPR as bio-inoculants is a key approach to sustainable agriculture [4,33]. Most isolates showed halo zones indicating phosphate solubilization, with GAC-118 showing the widest at 11 mm. As a result, phosphate-solubilizing microbes are commonly used to promote plant growth and enhance soil fertility, while also helping to reduce the rising costs associated with harmful phosphate fertilizers. *Pseudomonas* is particularly effective as a phosphate-solubilizing genus. These findings align with research identifying *Rhizobium, Azotobacter, Pseudomonas*, and *Bacillus* as phosphate solubilizers [21,22,30].

### 4.4 Molecular analysis of *ipdC, nifK,* and *nifH* genes

Even small amounts of IAA from PGPR can enhance plant growth by promoting cell division, elongation, and disease resistance [38]. PGPR synthesizes IAA through various pathways, including the indole-3-pyruvic acid pathway, the indole-3-acetamide pathway, the tryptophan side chain pathway, the tryptamine pathway, and the indole-3-acetonitrile pathway, all of which depend on the amino acid tryptophan [39]. PGPR synthesizes IAA mainly through the IPyA pathway, which relies on tryptophan and is found in *Bacillus, Enterobacter, Azospirillum, Rhizobium, Pseudomonas,* and *Arthrobacter* [9,12].

Understanding the molecular mechanisms of IAA production by microbial isolates presents challenges. Regulatory mechanisms governing gene expression in IAA biosynthesis pathways are not fully elucidated, impacting our grasp of how environmental cues and metabolic signals influence these pathways [40]. Genetic diversity among microbial species leads to variability in enzymes and pathways for IAA production, essential for comprehensive understanding [41]. Interactions between IAA-producing microbes and host plants, including modulation of hormone signaling and growth promotion, remain unclear [42]. Environmental factors like nutrient availability, pH, and temperature significantly influence IAA production and require further investigation [43].

The *ipdC* gene is crucial for IAA synthesis in the IPyA pathway, converting tryptophan to IAA via indole-3-pyruvate and indole-3-acetaldehyde [38]. Zhang et al. [9] identified *ipdC* in *Pseudomonas putida, Bacillus,* and *Azospirillum brasilense*. Mining functional genes enhances PGPR strains and supports research on their growth-promoting traits. It can be used to strengthen research on the growth-promoting characteristics of strains, supplement the metabolism of strains, and improve the application efficiency of PGPR in plants [38].

Nitrogen fixation is facilitated by the nitrogenase enzyme, encoded by genes such as *nifH*, *nifD*, and *nifK* [22]. The *nifH* gene, which encodes the nitrogenase reductase subunit, is widely sequenced and serves as a reliable marker for identifying nitrogen-fixing bacteria [22,34]. This study confirmed the molecular analysis of *nifH* and *nifK* genes in selected cultures, identifying beneficial microorganisms. These findings are consistent with previous studies documenting *nifH* genes in rhizosphere bacteria from various leguminous plants [22,23,35,36]*.* The *nifH* gene is widely recognized as a key functional marker for assessing nitrogen fixation potential [34]. Previous research by Dai et al. [25] also identified *nifK* genes in genera like *Methylococcus, Betaproteobacteria, Herbaspirillum,* and *Burkholderia* from acid mine drainage environments.

### 4.5 Greenhouse evaluation of bacterial isolates for plant growth promotion

Bacteria that synthesize IAA are valuable for promoting plant growth. This study shows significant differences in IAA production between treated and control groups (p ≤ 0.05), highlighting its role in enhancing plant growth. *Bacillus* and *Pseudomonas* species are PGPR that enhance plant growth through mechanisms such as IAA production, nutrient uptake, pathogen suppression, and improved disease resistance, contributing to increased biomass, root development, and yield in crops like chickpea, wheat, and tomatoes [37,44]. *Bacillus* species are known for their production of growth-promoting substances like IAA, which can improve root development and nutrient uptake. Additionally, *Bacillus* species produce siderophores that enhance iron availability to plants and secrete enzymes that degrade harmful soil pathogens, thereby promoting plant health. Studies have shown that *Bacillus* inoculation leads to increased biomass, root length, and overall vigor in crops like chickpeas, wheat, and soybeans [37]. For instance, *Bacillus subtilis* has been reported to stimulate the germination of seeds, resulting in faster growth and higher yields. On the other hand, Pseudomonas species, particularly Pseudomonas fluorescens, also play a critical role in enhancing plant growth through mechanisms like phosphate solubilization, nitrogen fixation, and production of antimicrobial compounds. *Pseudomonas* strains often colonize the rhizosphere, competing with harmful pathogens and reducing disease incidence in plants. These bacteria produce volatile organic compounds and phytohormones, such as auxins and cytokinins, that enhance root growth and plant resilience. Studies have shown that *Pseudomonas* inoculation in crops like tomatoes and wheat significantly improves plant growth, yield, and disease resistance [44]. Similar studies in wheat [11], maize [45], sorghum [10], mung [46], coffee [47], and chickpea [21] have also explored bacterial inoculation effects.

The treatment with GAC-118 resulted in the highest plant shoot height of 35.867 cm, compared to the un-inoculated control at 19.463 cm. These findings align with studies showing increased shoot height with selected isolates [10,17]. Birhanu et al. [33], who reported that all selected isolates increased shoot height compared to controls. The selected isolates significantly increased the shoot dry weight of chickpea seedlings. The potential of bacterial isolates to promote above-ground biomass growth varies based on genomic characteristics and factors like environmental conditions, soil type, and greenhouse conditions, as noted by Ref. [10]. The tested IAA-producing bacterial isolates demonstrated varying increases in shoot dry weight, consistent with [21].

Significant increases in plant root length were observed with bacterial isolate treatments compared to the un-inoculated control. This aligns with findings from Idris et al. [48] and Birhanu et al. [33], which also reported significant root length increases with different isolates. Isolates also showed significant increases in root fresh and dry weight compared to the control. However, not all isolates that increased root length also increased root fresh and dry weight in this study. Environmental conditions and variations in isolate characteristics may contribute to these discrepancies. In contrast, Yadav et al. [21] found that isolate' increasing root length also increased root fresh and dry weight, differing from our findings.

ANOVA results showed significantly higher agronomic parameter values (p ≤ 0.05) compared to the control. PGPR chickpea rhizosphere bacteria differed significantly in growth promotion, linked to their IAA production by Birhnau [10]. Strong and moderate Pearson correlation coefficients indicated positive relationships among agronomic parameters, consistent with prior studies by Birhanu et al. [33]. Anjum et al. [46] reported a negative correlation between plant height, root length, and fresh weight, contrasting this study's findings. Overall, these findings underscore IAA production's importance in effective bio-inoculants.

## 5 Conclusion

The confirmation of IAA production by PGPR isolated from the rhizosphere and soil samples of chickpea genotypes is a significant advancement. This research is particularly noteworthy for its focus on IAA production by PGPR from chickpea rhizosphere soil. The study contributes valuable insights into the auxin-producing PGPR that inhabit the chickpea rhizosphere. The highest IAA production was observed in *Bacillus* and *Pseudomonas* species cultured in a medium supplemented with 500 μg/ml of L-tryptophan at 35 °C, following a 72-h incubation period. The results promise to develop a formulation of potentially active plant growth-promoting strains that can promote plant growth and improve crop yields. In the absence of chemical fertilizers, genera such as *Pseudomonas* and *Bacillus* could serve as valuable alternatives for promoting plant growth and could be an excellent resource for developing biofertilizers for organic farming, especially in developing countries like Ethiopia. However, more research is required to confirm these potential isolates in detail and field application, considering different soil types and climates, particularly for crops that are significant economically and molecular identification through 16S rRNA sequencing.

## CRediT authorship contribution statement

**Debebe Landina Lata:** Writing – review & editing, Writing – original draft, Validation, Methodology, Investigation, Conceptualization. **Oumer Abdie:** Writing – review & editing, Visualization, Validation, Resources, Data curation. **Yayis Rezene:** Validation, Supervision, Resources, Funding acquisition.

## Ethics approval

No ethics problem.

## Data and code availability

Data will be made available on request.

## Declaration of generative AI and AI-assisted technologies

During the preparation of this review, I utilized the ChatGPT tool (version 3.5) for rewriting, paraphrasing, spell-checking, and generating updated ideas on the related topic. After using this tool/service, I thoroughly reviewed and edited the content as needed and took full responsibility for the final publication.

## Funding

This work was supported by 10.13039/501100022540Wolkite University, Wolkite, Ethiopia.

## Declaration of competing interest

The authors declare that they have no known competing financial interests or personal relationships that could have appeared to influence the work reported in this paper:**Debebe Landina Lata** reports financial support was provided by wolkite University, Department of Biotechnology. **Debebe Landina Lata** reports a relationship with 10.13039/501100022540Wolkite University that includes: employment. If there are other authors, they declare that they have no known competing financial interests or personal relationships that could have appeared to influence the work reported in this paper.
